# Supplementary material for: NetTCR-struc, a structure driven approach for prediction of TCR-pMHC interactions
Source: Front Immunol. 2025 Jul 17;16:1616328. doi: 10.3389/fimmu.2025.1616328 (PMC12311634; doi:10.3389/fimmu.2025.1616328)
Supplement: Supplementary file 1 [file Table1.docx]

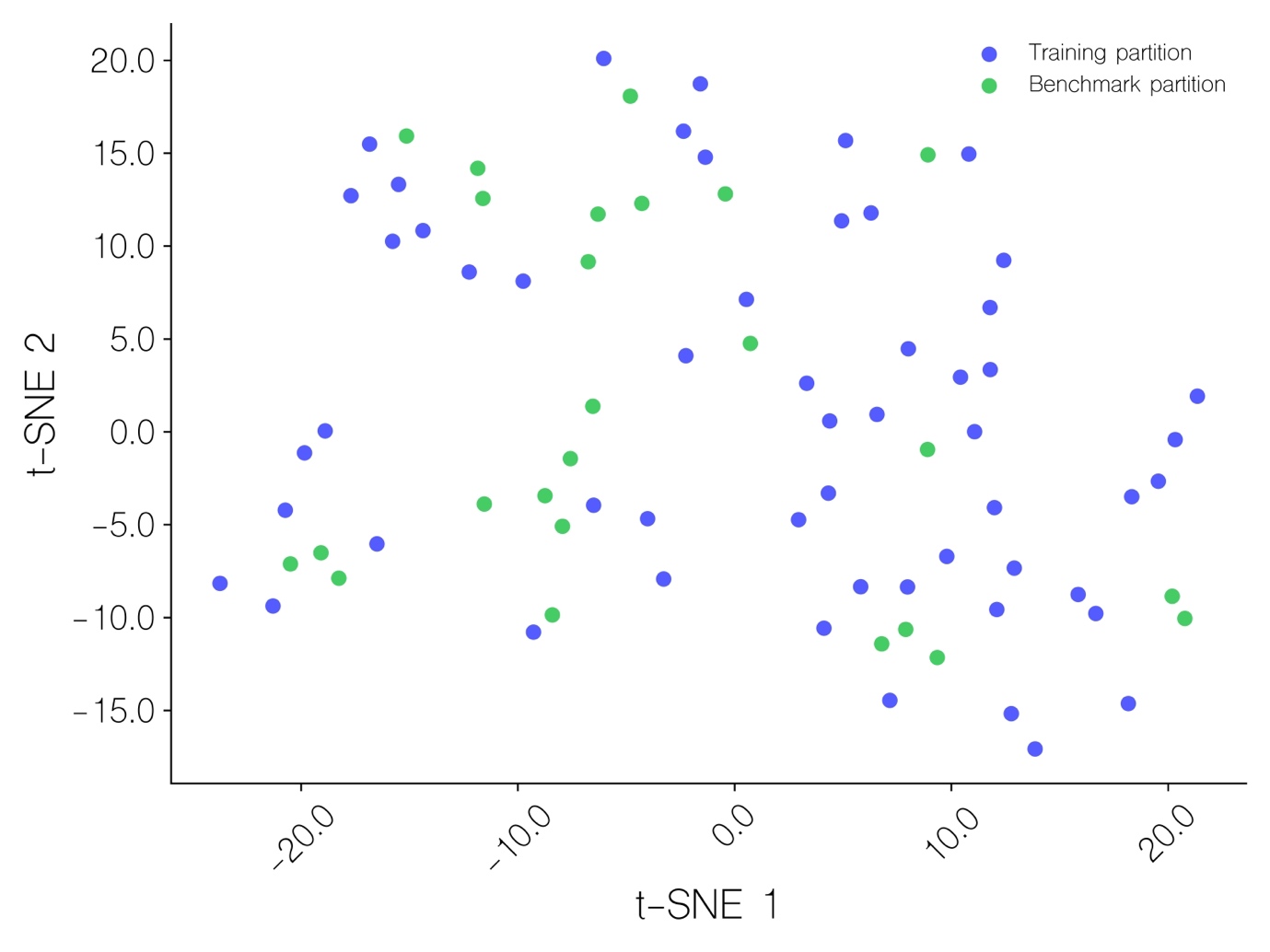


**Supplementary 1: t-SNE visualization of ESM-IF1 embeddings of structural models for the DockQ regression dataset.** For each target in the TCR-pMHC model quality assessment dataset, the top-ranked structural model was selected. Residues from the TCRα and TCRβ chains of these models were then embedded using the ESM-IF1 inverse folding model (for more information, refer to materials and methods). The embedded chains were subsequently mean-pooled along the sequence dimension, and the two resulting vectors were concatenated, yielding vectors of length 1024 representing each TCR. The structural embeddings were projected into two dimensions using t-distributed stochastic neighbor embedding (t-SNE), as implemented in scikit-learn v1.0.2, with a perplexity of 10 and a fixed random seed of 0. Training and benchmark data TCRs appear similarly scattered, indicating little redundancy and biases between the two datasets.

**
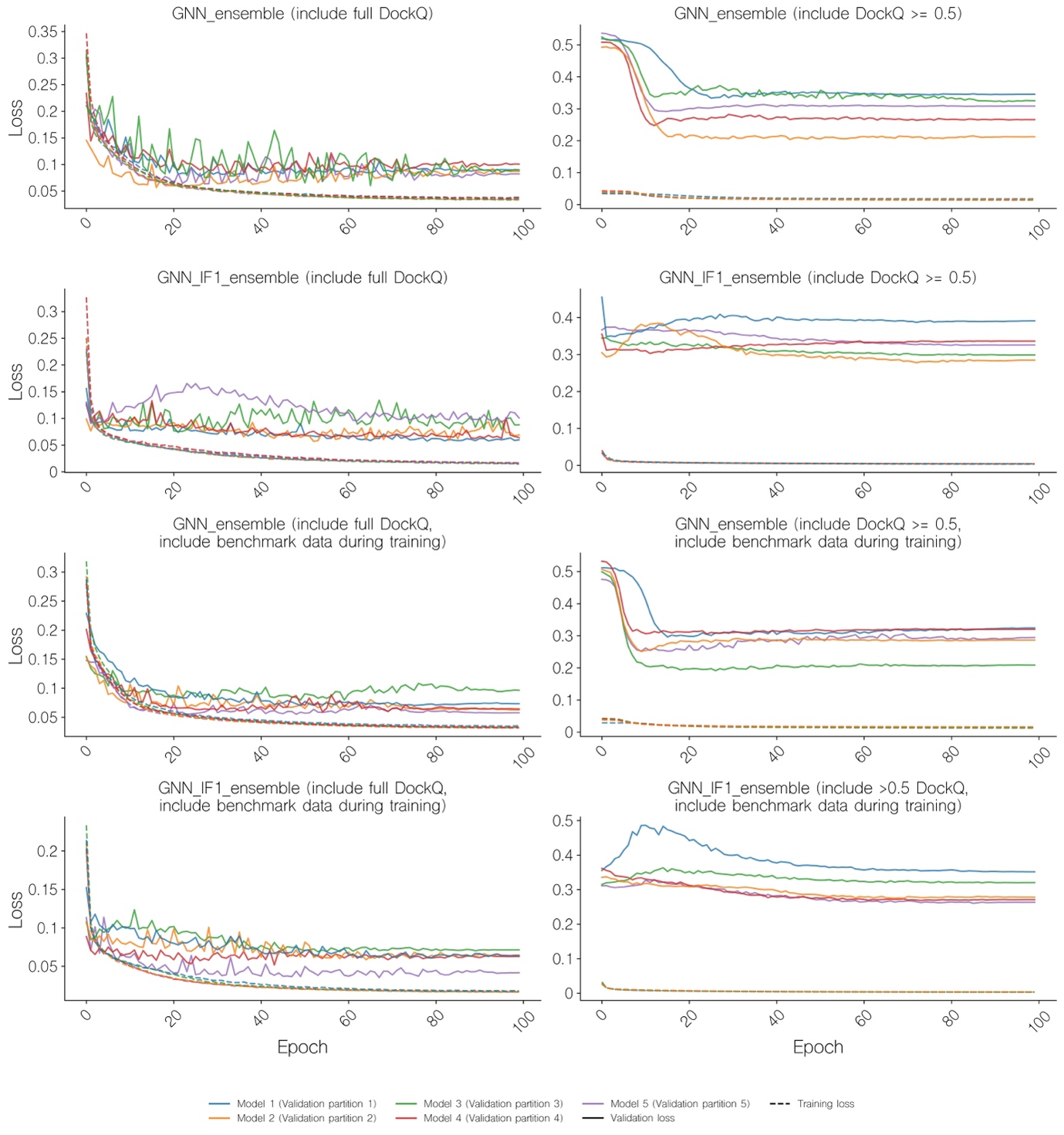
 Supplementary 2: Visualization of training and validation loss during model training.** GVP-GNN models were trained to predict DockQ scores for TCR-pMHC class I structural models using 5-fold cross-validation setups. Loss was computed using a pair MSE loss function. For more information on how these partitions were generated and details on the loss function, refer to the materials and methods section. Models were trained on two datasets, one composed only of structural models with a DockQ score over 0.5, and one spanning the full range of the DockQ metric. In all cases, validation loss was computed on the full dataset. Consequently, the validation loss reported for all >0.5 DockQ models (right column) is substantially higher than the training loss, as these models are not trained to predict DockQ for the <0.5 DockQ structures present in the validation sets. In all cases, the models converge on low training loss solutions (Pair MSE ≈ 0.01). For the models trained on the full DockQ datasets (left column), validation loss converges on Pair MSE ≈ 0.1 as expected without signs of noticeable overfitting.


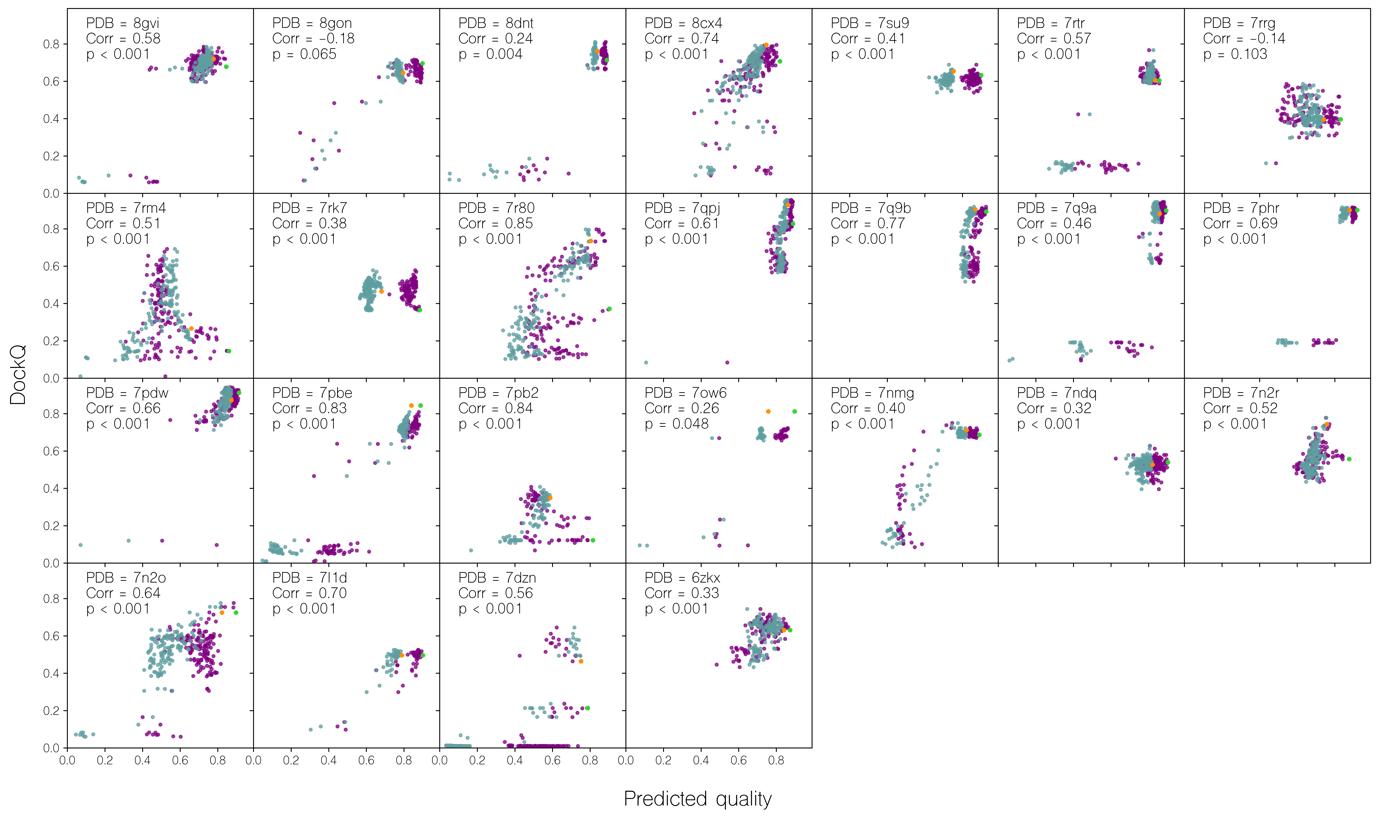


**Supplementary 3: Per-benchmark target correlation of DockQ and predicted quality.** Green: GNN-AF. Purple: AF_confidence. Orange and green dots indicate the highest confidence for each target, i.e. the candidate that would be selected as top 1 for GNN-AF and AF_confidence, respectively. The Spearman correlation coefficient for GNN-AF and DockQ is shown in each subplot.


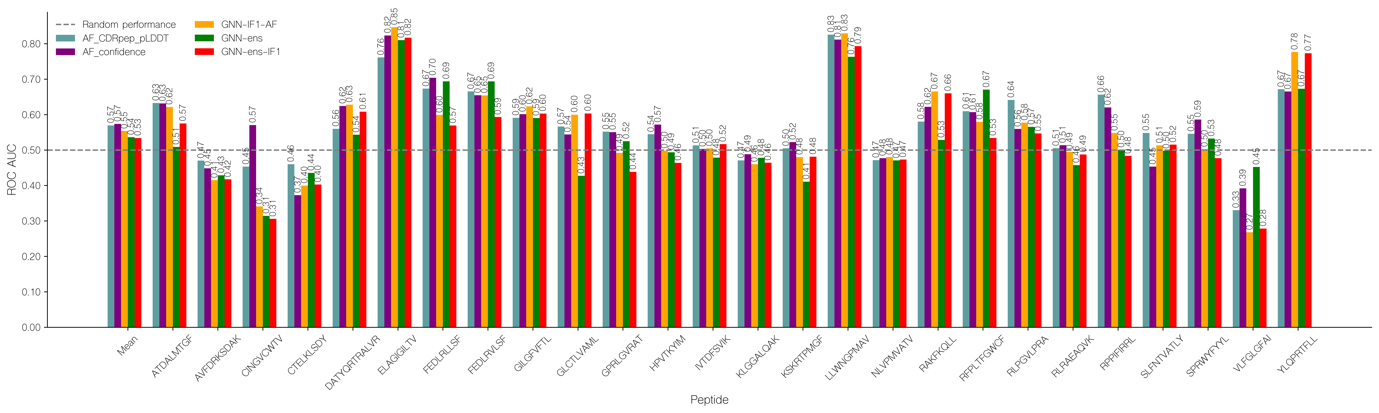


**Supplementary 4: TCR-pMHC binding classification ROC AUC for different scoring methods.** The different methods are: AF_confidence: AlphaFold-M confidence, AF_CDRpep_pLDDT: Mean pLDDT of CDR123 and peptide residues, GNN-ens: GVP-GNN ensemble, GNN-ens-IF1: GVP-GNN ensemble trained including ESM-IF1 structural embeddings. GNN-IF1-AF: Harmonic mean of GNN-ens-IF1, AF_confidence and AF_CDRpep_pLDDT. For more information on the GVP-GNN ensembles, see text and materials and methods.


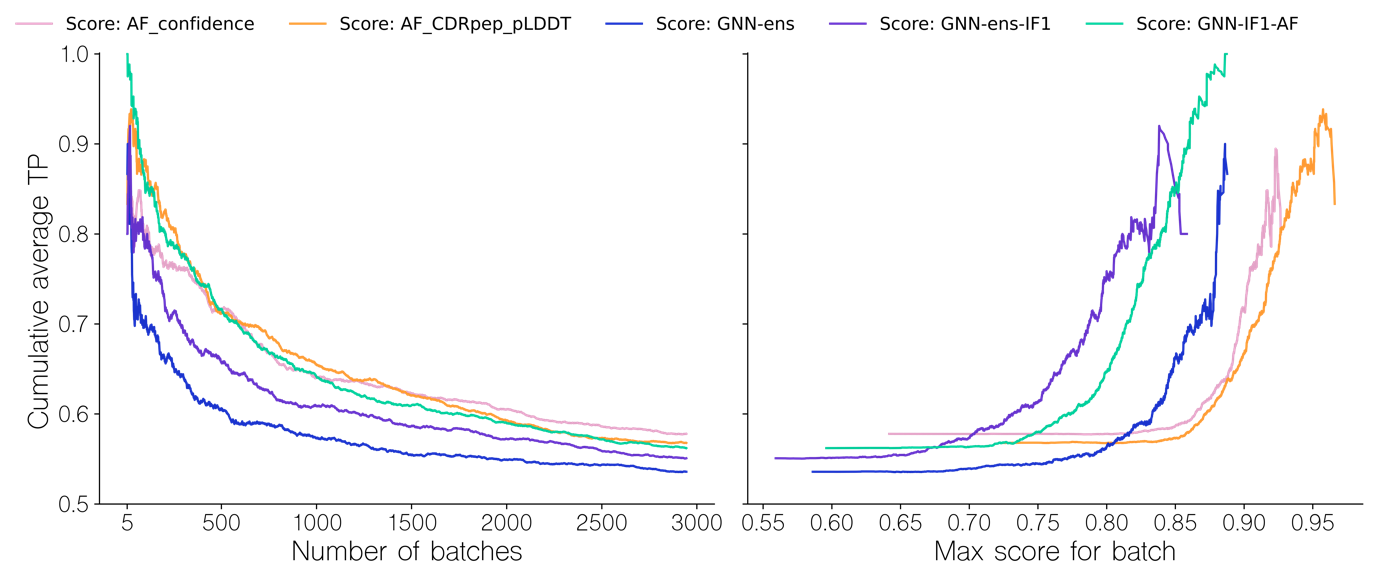


**Supplementary 5: Performance evaluation of the different methods in the batch evaluation when selecting top 1 based on GNN-AF.** Left (a): Cumulative TPR curve for batches sorted by descending maximal intra-batch quality score for a given method. The TPR value for a given batch is computed from the ranking of the binding complex within each batch, based on their predicted quality, with a value of 1 corresponding to a top 1 rank. Right (b): Cumulative TPR curve for batches as a function of the maximal intra-batch quality score. In both plots, docking candidate selection was performed using only the GNN-AF score. The different methods are: AF_confidence: AlphaFold-M confidence, AF_CDRpep_pLDDT: Mean pLDDT of CDR123 and peptide residues, GNN-ens: GVP-GNN ensemble, GNN-ens-IF1: GVP-GNN ensemble trained including ESM-IF1 structural embeddings. GNN-IF1-AF: Harmonic mean of GNN-ens-IF1, AF_confidence and AF_CDRpep_pLDDT. For more information on the GVP-GNN ensembles, see text and materials and methods.
